# A Study of Awareness, Knowledge, Attitudes, and Practices Regarding Antibiotic Resistance

**DOI:** 10.7759/cureus.62854

**Published:** 2024-06-21

**Authors:** Anas Alhur, Lama Alghamdi, Fay Alqahtani, Milaf Alshammari, Halimah Hattany, Abdulrahman Akshah, Atyaf Al Ahmary, Rawan Aloqran, Ebtehal Olagi, Noura S Alshahrani, Reem Al-Qahtani, Joud Alqahtani, Lena Alghamdi, Abdullah Alharbi, Shahad Alshokani

**Affiliations:** 1 Health Informatics, University of Hail, College of Public Health and Health Informatics, Hail, SAU; 2 Clinical Pharmacy, College of Pharmacy, Al-Baha University, Al-Baha, SAU; 3 Clinical Pharmacy, College of Pharmacy, King Khalid University, Abha, SAU; 4 Clinical Pharmacy, College of Pharmacy, University of Hafr Albatin, Hafr Albatin, SAU; 5 Clinical Pharmacy, College of Pharmacy, Jazan University, Jazan, SAU; 6 Clinical Pharmacy, College of Pharmacy, University of Hafar Albatin, Hafar Albatin, SAU; 7 Laboratory Medicine, Armed Forces Hospitals Southern Region (AFHSR), Khamis Mushait, SAU; 8 Clinical Pharmacy, King Khalid University, Abha, SAU

**Keywords:** educational interventions, demographic analysis, practices, attitudes, knowledge, public health, antibiotic resistance

## Abstract

Background

Antibiotic resistance is a critical global health concern, intensified by public misconceptions and inconsistent antibiotic use. Misunderstandings about antibiotics and their improper use contribute to the acceleration of resistance, making it harder to treat infections effectively. Previous research has identified knowledge gaps in the public, yet there is limited understanding of how these gaps translate into attitudes and practices across different demographic groups. This study aimed to assess the levels of knowledge, attitudes, and practices regarding antibiotic resistance among various demographic groups and to determine the influence of demographic factors on these variables.

Methods

A descriptive study was conducted using a structured online questionnaire distributed through social media and health forums. The questionnaire targeted adults aged 18 years and older from diverse socioeconomic backgrounds. Data were analyzed using SPSS version 26 (Armonk, NY: IBM Corp.) for descriptive statistics, chi-square tests, and logistic regression analysis.

Results

The study included responses from 1,561 participants, revealing that 75.72% had knowledge of antibiotic resistance, but only 68.23% understood its public health implications. Attitudes toward antibiotic use were generally positive, with 90.14% recognizing the importance of completing antibiotic courses. However, 32.16% admitted they would stop taking antibiotics once feeling better, even if the course was not finished, highlighting a significant gap between knowledge and practice. Regression analysis identified awareness of prevention measures (coefficient=2.06) and knowledge of antibiotic resistance (coefficient=1.38) as strong predictors of awareness. The chi-square test showed a significant association between gender and awareness of prevention measures (chi-square value=15.19, p-value=0.000097).

Conclusions

Despite a high level of knowledge about antibiotic resistance, gaps in attitudes and practices persist. These findings underscore the necessity for tailored educational campaigns that not only inform but also engage and alter behaviors across all demographic groups to combat antibiotic resistance effectively.

## Introduction

Antibiotics have revolutionized modern medicine, saving countless lives by effectively treating bacterial infections. However, the growing problem of antibiotic resistance threatens to undermine these critical medications. Antibiotic resistance occurs when bacteria evolve to survive exposure to antibiotics, rendering these drugs less effective or even useless [1]. This issue presents a grave challenge to global health, leading to serious clinical and economic consequences. The problem is further aggravated by widespread public misunderstandings and inconsistent practices surrounding antibiotic use, which can accelerate the emergence and propagation of resistance [2,3]. This study aimed to examine current knowledge gaps, attitudes, and practices related to antibiotic use among the general population, highlighting the urgent need for targeted educational interventions.

Misconceptions and knowledge gaps about the necessity and proper usage of antibiotics contribute significantly to the issue of resistance. For instance, many individuals erroneously believe that antibiotics are effective against viral infections, leading to inappropriate usage [4]. Numerous studies have revealed significant gaps in public knowledge about antibiotic resistance across various demographic groups. Research by Napolitano et al. indicates that knowledge about antibiotic resistance is weakly correlated with demographic factors, highlighting the need for comprehensive and targeted educational approaches [5]. Similarly, Mason et al. emphasized a general lack of awareness, underscoring the critical necessity for enhanced educational outreach [6]. Misinformation about antibiotics is a worldwide problem, with studies like Kim et al. in South Korea showing common misconceptions even among well-educated populations [7]. Addressing these knowledge gaps is crucial for promoting proper antibiotic use and curbing the spread of resistance.

Misuse and non-adherence of antibiotics, such as non-adherence to prescribed regimens, pose substantial risks and significantly contribute to the development of resistance. Ventola examined these risks, showing that behaviors like stopping antibiotics early or using leftover medications can lead to the survival of resistant bacteria, exacerbating the problem [8]. Other notable contributions include Dyar et al., who investigated antibiotic use in outpatient settings across Europe, and Barah and Gonçalves, who reported on the misuse of antibiotics in the Syrian Arab Republic [9,10]. These studies illustrate the global nature of the problem and the diverse factors influencing antibiotic practices.

This study aims to address the following research questions: (1) What is the current level of knowledge about antibiotic resistance among various demographic groups? (2) How do attitudes toward antibiotic use vary across the population? (3) What practices are prevalent regarding the use and disposal of antibiotics among the general population?

## Materials and methods

Study design

This study employed a quantitative, cross-sectional design using an online questionnaire to assess public knowledge, attitudes, and practices related to antibiotic use and resistance in Saudi Arabia. The questionnaire was developed based on a comprehensive literature review and incorporated both original questions and adapted items from validated instruments, such as the antibiotic knowledge and awareness questionnaire (AKAQ) [11]. To ensure content validity, the questionnaire underwent expert review by a panel of healthcare professionals and researchers in the field of antibiotic resistance, and their feedback was incorporated to refine the questions and improve the overall clarity and relevance of the instrument.

Population and sample

The target population for this study included adults aged 18 years and older residing in Saudi Arabia, with a focus on diverse socioeconomic and educational backgrounds to ensure a comprehensive understanding of community-wide antibiotic use practices. A convenience sampling method was employed to recruit participants due to its efficiency in reaching a large audience within a relatively short timeframe. Although this non-probability sampling approach may limit the generalizability of the findings, efforts were made to recruit participants from various regions and backgrounds to mitigate this limitation. The sample size was determined using G*Power software (Düsseldorf, Germany: Heinrich Heine University), considering a medium effect size (0.3), a power of 0.80, and an alpha level of 0.05 for chi-square tests, resulting in a minimum required sample size of 143 participants [12]. However, to account for potential incomplete responses and ensure adequate representation across demographic groups, the study aimed to recruit a larger sample of at least 1,000 participants.

Data collection

The online questionnaire was distributed through popular social media platforms, including Facebook, Twitter, and Instagram, over a period of three months from March 2, 2024, to May 18, 2024. The researchers collaborated with several universities, such as the University of Hail (UOH), King Abdulaziz University (KAU), and King Saud University (KSU) to share the survey invitation with their students and staff. Additionally, community health organizations, including the Saudi Patient Safety Center (SPSC), assisted in disseminating the questionnaire to their networks. To encourage participation, the questionnaire was designed to be concise and user-friendly, taking approximately 5-10 minutes to complete. Participants were informed that their responses would contribute to a better understanding of antibiotic use and resistance, emphasizing the importance of their participation. Reminder emails were sent to potential participants one and two weeks after the initial invitation to maximize the response rate.

Data analysis

The collected data were analyzed using SPSS version 26 (Armonk, NY: IBM Corp.). Descriptive statistics, including frequencies and percentages, were calculated to summarize the demographic characteristics and survey responses. Chi-square tests were used to examine associations between categorical variables, such as demographic factors and knowledge levels, as these tests are appropriate for analyzing the relationship between two categorical variables [13]. Logistic regression analysis was employed to identify predictors of antibiotic misuse, as this method is suitable for modeling the relationship between a binary outcome variable and one or more predictor variables [4]. Out of the 1,500 individuals invited to participate, 1,200 completed the questionnaire, resulting in a response rate of 80%. To assess potential non-response bias, the demographic characteristics of the respondents were compared to those of the target population using chi-square tests, and no significant differences were found, suggesting that non-response bias may not have had a substantial impact on the results.

Ethical considerations

Ethical approval for this study was obtained from the Research Ethics Committee (REC) of the University of Hail, Saudi Arabia, with protocol approval number H-2024-338. All participants provided informed consent, and their confidentiality and privacy were strictly maintained by anonymizing personal identifiers in the dataset. Participation in the study was voluntary, and participants could withdraw at any time without consequences.

## Results

The study revealed a diverse age distribution among participants. The largest group comprised individuals aged 18-24 years, who represented 39.14% of the sample (n=611). This was followed by those aged 25-34 years and 45-54 years, with frequencies of 260 (16.66%) and 257 (16.46%), respectively. Participants aged 55-64 years accounted for 13.32% (n=208), while those in the 35-44 years age group made up 12.68% (n=198). The smallest group was individuals aged 65 years and older, contributing to only 1.73% of the total (n=27).

Regarding gender, females constituted a majority of the respondents, totaling 68.48% (n=1,069). Males were less represented, comprising 31.52% of the sample (n=492). In terms of education levels among participants, the majority reported having attended college or university, making up 76.43% of respondents (n=1,193). This was significantly higher compared to other educational groups. Participants with a high school education were 13.77% (n=215), those with post-graduate qualifications were 7.11% (n=111), and a smaller fraction reported having less than a high school education at 2.69% (n=42) (Table 1).

**Table 1:** Demographic information of participants.

Category	Group	Frequency	Percentage
Q1: Age distribution (years)	18-24	611	39.14%
	25-34	260	16.66%
	45-54	257	16.46%
	55-64	208	13.32%
	35-44	198	12.68%
	65 and older	27	1.73%
Q2: Gender distribution	Female	1,069	68.48%
	Male	492	31.52%
Q3: Education level	College/university	1,193	76.43%
	High school	215	13.77%
	Post-graduate	111	7.11%
	Less than high school	42	2.69%

The results assessed participants' knowledge about antibiotic resistance. A majority, 75.72% (n=1,182), indicated that they understood what antibiotic resistance is, while 24.28% (n=379) did not. Participants were asked if they were aware that antibiotic resistance could affect anyone regardless of age or gender. A significant portion, 82.45% (n=1,287), responded affirmatively, demonstrating a high level of awareness, whereas 17.55% (n=274) were not aware.

When questioned on the primary causes of antibiotic resistance, 83.34% (n=1,301) believed it was primarily due to the overuse of antibiotics. Conversely, 16.66% (n=260) disagreed with this view. Knowledge of the consequences of antibiotic resistance on public health was evident in 68.23% (n=1,065) of the participants, while 31.77% (n=496) were unaware of the implications. Also, there was a near-even split in responses regarding measures to prevent antibiotic resistance. A slight majority, 52.08% (n=813), were not aware of any measures, while 47.92% (n=748) were informed about preventive strategies (Table 2).

**Table 2:** Knowledge about antibiotic resistance.

Question	Response	Frequency	Percentage
Q4: Do you know what antibiotic resistance is?	Yes	1,182	75.72%
	No	379	24.28%
Q5: Are you aware that antibiotic resistance can affect anyone regardless of age or gender?	Yes	1,287	82.45%
	No	274	17.55%
Q6: Do you believe antibiotic resistance is primarily caused by the overuse of antibiotics?	Yes	1,301	83.34%
	No	260	16.66%
Q7: Do you know the consequences of antibiotic resistance on public health?	Yes	1,065	68.23%
	No	496	31.77%
Q8: Are you aware of any measures to prevent antibiotic resistance?	No	813	52.08%
	Yes	748	47.92%

A strong majority of respondents (90.14% combined for "strongly agree" and "agree") recognize the importance of completing a prescribed antibiotic course. This high level of agreement underscores a general acknowledgment of completing treatments as essential to preventing the development of resistance. Despite understanding the importance of completing antibiotic courses, 32.16% of participants admitted they would stop taking antibiotics once they feel better, even if the course is not finished. This behavior could potentially contribute to antibiotic resistance, highlighting a gap between knowledge and practice.

Responses varied regarding trust in healthcare professionals with 45.23% stating they often trust and 19.80% always trust their prescriptions. However, 28.96% only sometimes trust these prescriptions, indicating some skepticism or uncertainty about the appropriateness of prescribed antibiotics. A significant portion of the sample (64.77% combined for "somewhat concerned" and "very concerned") expressed concern about antibiotic resistance, indicating awareness of its seriousness. However, a non-trivial minority remains less concerned or neutral, which could impact public health efforts. A combined 65.96% would sometimes or often accept an antibiotic prescription without questioning its necessity, reflecting a trust in medical advice but also a potential for accepting unnecessary antibiotics (Table 3).

**Table 3:** Attitudes towards antibiotic use and resistance.

Question	Response	Frequency	Percentage
Q9: Do you think it is important to complete an antibiotic course prescribed by a healthcare provider?	Strongly agree	1,011	64.77%
	Agree	396	25.37%
	Neutral	122	7.82%
	Disagree	23	1.47%
	Strongly disagree	9	0.58%
Q10: Would you stop taking antibiotics once you feel better, even if the prescribed course is not finished?	No	1,059	67.84%
	Yes	502	32.16%
Q11: Do you trust that healthcare professionals are prescribing antibiotics appropriately?	Often	706	45.23%
	Sometimes	452	28.96%
	Always	309	19.80%
	Rarely	67	4.29%
	Never	27	1.73%
Q12: How concerned are you about the issue of antibiotic resistance?	Somewhat concerned	660	42.28%
	Neutral	388	24.86%
	Very concerned	351	22.49%
	Not very concerned	144	9.22%
	Not concerned at all	18	1.15%
Q13: Would you accept a prescription for antibiotics without questioning its necessity?	Sometimes	538	34.47%
	Often	483	30.94%
	Always	274	17.55%
	Rarely	168	10.76%
	Never	98	6.28%

Approximately one-third (33.70%) of respondents admitted to taking antibiotics that were prescribed for someone else, a practice that can be risky and contribute to inappropriate antibiotic use. Most participants (78.92%) claimed they always consult a healthcare professional before taking antibiotics, which is a positive practice in preventing misuse and overuse.

A significant minority (26.46%) reported taking antibiotics without a prescription in the past year, highlighting a concerning behavior in terms of self-medication and potential misuse. Over a third (36.32%) keep leftover antibiotics for future use, a practice that can lead to inappropriate use and increased resistance. Nearly 69.53% (combining "always" and "often") actively seek information about the correct use of antibiotics from reliable sources, indicating a proactive approach to informed medication practices (Table 4).

**Table 4:** Practices related to antibiotic use.

Question	Response	Frequency	Percentage
Q14: Have you ever taken antibiotics that were prescribed for someone else?	No	1035	66.30%
	Yes	526	33.70%
Q15: Do you always consult a healthcare professional before taking antibiotics?	Yes	1232	78.92%
	No	329	21.08%
Q16: In the past year, have you taken antibiotics without a prescription?	No	1148	73.54%
	Yes	413	26.46%
Q17: Do you usually keep leftover antibiotics for future use?	No	994	63.68%
	Yes	567	36.32%
Q18: Do you seek information about the correct use of antibiotics from reliable sources?	Always	702	44.97%
	Often	386	24.73%
	Sometimes	294	18.83%
	Rarely	105	6.73%
	Never	74	4.74%

The results also indicate a good foundational understanding of antibiotic resistance among respondents. The mean response for awareness of what antibiotic resistance is was 0.76, with a standard deviation (SD) of 0.43, demonstrating that a majority recognize the concept. Awareness that antibiotic resistance can affect anyone regardless of age or gender is higher, with a mean of 0.82 and an SD of 0.38, reflecting a broad understanding of its universal risk. The belief that the overuse of antibiotics primarily causes antibiotic resistance had a mean of 0.83 and an SD of 0.37, indicating strong agreement on this cause among participants. However, knowledge of the public health consequences of antibiotic resistance was lower, with a mean of 0.68 and an SD of 0.47, suggesting that the implications of antibiotic resistance are less well understood. Awareness of measures to prevent antibiotic resistance was notably lower, with a mean of 0.48 and an SD of 0.50, highlighting a significant area for educational improvement.

Attitudes towards antibiotic usage revealed that a large proportion of respondents value completing antibiotic courses as prescribed. The importance of completing treatment had a high mean of 1.94 with an SD of 1.32. However, a substantial proportion might discontinue treatment early once feeling better, with a mean of 0.32 and an SD of 0.47. Trust in the appropriateness of antibiotic prescriptions by healthcare professionals had a mean of 3.77 and an SD of 0.87, showing that most respondents generally trust medical advice. Concern about antibiotic resistance also scored high, with a mean of 3.76 and an SD of 0.94, yet the willingness to accept prescriptions without questioning had a mean of 3.43 and an SD of 1.09, indicating varied levels of critical engagement with healthcare providers.

Practical behaviors concerning antibiotic usage among respondents showed some areas of concern. The practice of using antibiotics prescribed for someone else had a mean of 0.34 and an SD of 0.47. Consulting healthcare professionals before taking antibiotics had a mean of 0.79 and an SD of 0.41, suggesting that most respondents seek professional advice. However, the incidence of taking antibiotics without a prescription in the past year had a mean of 0.26 and an SD of 0.44. The habit of keeping leftover antibiotics for future use also presented a risk, with a mean of 0.36 and an SD of 0.48. Lastly, the frequency of seeking information about the correct use of antibiotics from reliable sources was relatively high, with a mean of 3.98 and an SD of 1.16, indicating a proactive approach by many to educate themselves as demonstrated in Table 5.

**Table 5:** Descriptive statistics (mean and SD) for knowledge, attitudes, and practices related to antibiotic use and resistance.

Section	Question description	Mean	Standard deviation
Section 2: knowledge			
Knowledge of antibiotic resistance	Do you know what antibiotic resistance is?	0.76	0.43
Awareness of universal risk	Are you aware that antibiotic resistance can affect anyone, regardless of age or gender?	0.82	0.38
Causes of resistance	Do you believe antibiotic resistance is primarily caused by the overuse of antibiotics?	0.83	0.37
Consequences of resistance	Do you know the consequences of antibiotic resistance on public health?	0.68	0.47
Prevention measures awareness	Are you aware of any measures to prevent antibiotic resistance?	0.48	0.5
Section 3: attitudes			
Importance of completing treatment	Do you think it is important to complete an antibiotic course prescribed by a healthcare provider?	1.94	1.32
Stopping treatment early	Would you stop taking antibiotics once you feel better, even if the prescribed course is not finished?	0.32	0.47
Trust in prescriptions	Do you trust that healthcare professionals are prescribing antibiotics appropriately?	3.77	0.87
Concern about resistance	How concerned are you about the issue of antibiotic resistance?	3.76	0.94
Questioning prescriptions	Would you accept a prescription for antibiotics without questioning its necessity?	3.43	1.09
Use of others' antibiotics	Have you ever taken antibiotics that were prescribed for someone else?	0.34	0.47
Consulting professionals	Do you always consult a healthcare professional before taking antibiotics?	0.79	0.41
Antibiotics without prescription	In the past year, have you taken antibiotics without a prescription?	0.26	0.44
Keeping leftover antibiotics	Do you usually keep leftover antibiotics for future use?	0.36	0.48
Seeking reliable information	Do you seek information about the correct use of antibiotics from reliable sources?	3.98	1.16

The logistic regression model used to predict awareness of the public health consequences of antibiotic resistance performed well, achieving an overall accuracy of 82%. This indicates that the model is effective in predicting whether individuals are aware of the consequences of antibiotic resistance. The model's precision for "no" predictions was 72%, meaning that 72% of all "no" predictions were correct, while for "yes" predictions, the precision was 87%, indicating that 87% of all "yes" predictions were accurate. The recall, or the model's ability to identify all relevant cases, was 71% for "no" and 87% for "yes," suggesting that the model was particularly effective at identifying "yes" responses. The F1 score, which balances precision and recall, was 0.72 for "no" and 0.87 for "yes," reflecting strong model performance, especially in predicting "yes" outcomes (Table 6).

**Table 6:** Logistic regression summary.

Metric	Value
Accuracy	82%
Precision (no)	0.72
Precision (yes)	0.87
Recall (no)	0.71
Recall (yes)	0.87
F1 score (no)	0.72
F1 score (yes)	0.87

The coefficients of the logistic regression model indicate how various predictors influence the likelihood of being aware of the public health consequences of antibiotic resistance. Positive coefficients increase the likelihood, while negative coefficients decrease it. For example, the presence of awareness of prevention measures has a significant positive impact, with a coefficient of 2.06, indicating a strong influence on increasing awareness. Additionally, we have provided the odds ratios, confidence intervals, and p-values along with the coefficients for a comprehensive understanding (Table 7).

**Table 7:** Logistic regression summary.

Predictor	Coefficient	Odds ratio	95% confidence interval	p-Value
Awareness of prevention measures	2.06	7.87	5.89-10.52	<0.001
Awareness that antibiotic resistance can affect anyone	1.41	4.1	3.22-5.23	<0.001
Knowledge of what antibiotic resistance is	1.38	3.97	3.12-5.05	<0.001
Belief that antibiotic resistance is caused by overuse of antibiotics	0.87	2.39	1.87-3.05	<0.001
Education level (less than high school)	0.49	1.63	1.12-2.36	0.012
Age group 55-64 years	0.35	1.42	1.03-1.96	0.033
Consulting a healthcare professional before taking antibiotics	0.24	1.27	0.99-1.63	0.059
Age group 18-24 years	-0.47	0.63	0.49-0.80	<0.001

The chi-square test was conducted to examine if there is a statistically significant association between gender and awareness of measures to prevent antibiotic resistance. The test yielded a chi-square value of 15.19, indicating a significant difference between observed and expected frequencies. The very low p-value of 0.000097 strongly suggests that the relationship observed is not due to chance, hence confirming that gender and awareness are indeed related. The test involved one degree of freedom, appropriate for a 2x2 contingency table analysis (Table 8).

**Table 8:** Chi-square test of independence (gender vs. awareness of prevention measures).

Description	Value
Chi-square value	15.19
P-value	0.000097
Degrees of freedom	1

The contingency table for this analysis showed the distribution of responses by gender, where females and males were coded as 0 and 1, respectively. The responses were categorized into "not aware" and "aware." The table provided the counts for each category, which were critical for conducting the chi-square test. Females showed a higher number of "not aware" responses compared to males, who had a higher proportion of "aware" responses (Table 9).

**Table 9:** Contingency table for gender vs. awareness.

Gender (male=1, female=0)	Not aware (0)	Aware (1)
Female	593	476
Male	220	272

## Discussion

The findings from this study are consistent with a substantial body of research indicating that public understanding of antibiotic resistance, while relatively informed, often fails to translate into appropriate behavior. For example, McCullough et al. discovered that even when people recognize the issue of antibiotic resistance, they frequently fail to adhere to practices that mitigate its risk [7]. Similarly, a survey by Napolitano et al. highlighted the disparity between knowledge and behavior regarding antibiotic use in Italy [1]. Comparing our results with similar studies conducted in Saudi Arabia, such as those by Alhur et al. and Hawkins et al., reveals consistent patterns of knowledge gaps and misuse of antibiotics [14,15]. These findings underscore the need for targeted interventions that address both informational and behavioral aspects of antibiotic use.

The demographic data in our study, characterized by a predominance of younger adults and females with higher education levels, is in line with findings by Zajmi et al., who reported that younger, educated individuals often exhibit better knowledge but not necessarily better practices [16]. This suggests that interventions need to be multifaceted, addressing both informational and behavioral components. However, it is crucial to consider how factors, such as affordability, access to healthcare, and health literacy, might impact antibiotic practices. In Saudi Arabia, disparities in healthcare access and affordability, particularly among rural and lower-income populations, may contribute to the inappropriate use of antibiotics [17]. Limited health literacy can also hinder the understanding and adherence to proper antibiotic use guidelines [18].

Participants' willingness to discontinue antibiotic treatment upon feeling better is particularly concerning and reflects a common misconception reported in multiple studies [5,10]. Despite understanding the necessity to complete treatment courses, the immediate relief of symptoms often leads to non-compliance, a key driver of resistance development. Sociocultural factors, such as the pressure to return to work or school quickly, may also contribute to the premature discontinuation of antibiotic treatment [8].

As our results reveal, the practice of using antibiotics without a prescription has been identified as a significant problem in various regions. Studies by Kim et al. and Barah and Gonçalves both emphasize that non-prescribed antibiotic use is prevalent and linked to higher rates of antibiotic resistance [3,6]. In Saudi Arabia, the ease of accessing antibiotics without a prescription, coupled with cultural norms that favor self-medication, may exacerbate this issue [19]. Some respondents' alarming admission of using antibiotics prescribed for others has been noted in other contexts as well, such as the work by Currie et al., which confirms that such behavior is a global issue contributing to inappropriate antibiotic use and the spread of resistance [11].

Public health campaigns should, therefore, not only provide information but also aim to modify behavior, as suggested by Lecky et al., who found interactive educational approaches to be effective [12-14]. Similarly, interventions by Norris et al. that focused on behavioral changes showed promise in improving antibiotic use practices. Moreover, educational strategies should consider cultural and socioeconomic factors that influence antibiotic use practices, as noted by Hawkins et al. and Alhur et al., emphasizing tailored approaches to different demographic segments [15-19]. For instance, in Saudi Arabia, educational interventions should account for the influence of family dynamics, religious beliefs, and trust in healthcare providers on antibiotic use behaviors [20-23].

Limitations

While this study provides valuable insights into the knowledge, attitudes, and practices related to antibiotic resistance among the Saudi Arabian population, it is important to acknowledge its limitations. Firstly, the use of an online survey for data collection may have introduced sampling biases, as it primarily reaches individuals with internet access and those active on social media platforms. This may have underrepresented certain demographic groups, such as older adults, rural populations, or those from lower socioeconomic backgrounds, limiting the generalizability of the findings to the entire population.

Additionally, the study relied on self-reported data, which is subject to social desirability bias, where participants may have provided responses they perceived as more acceptable rather than reflecting their true beliefs and practices. Participants might have underreported their misuse of antibiotics or overreported adherence to proper practices to present themselves in a more favorable light. The study also did not assess the influence of healthcare provider counseling on participants' antibiotic use behaviors, which could be a significant factor in shaping these practices.

Moreover, the cross-sectional nature of the study captures data at a single point in time, limiting the ability to establish causal relationships between variables. Future research could employ longitudinal designs to better understand how knowledge, attitudes, and practices evolve over time and in response to interventions. The study also did not explore the potential influence of other factors, such as access to healthcare, insurance status, or the role of healthcare providers in shaping antibiotic use behaviors, which could be important areas for further investigation.

Despite these limitations, the current study provides a valuable foundation for understanding the complex interplay between knowledge, attitudes, and practices related to antibiotic resistance in Saudi Arabia. The insights gained can inform the development of targeted educational interventions and public health policies aimed at promoting responsible antibiotic use and mitigating the spread of resistance. Future research should build upon these findings, employing mixed-methods approaches, more representative sampling strategies, and objective measures of behavior to further elucidate the factors driving antibiotic misuse and resistance in the population.

## Conclusions

This study highlights a critical disparity between the general population's knowledge and their practices concerning antibiotic use in Saudi Arabia, demonstrating that while participants generally have a good understanding of antibiotic resistance, this knowledge often fails to translate into appropriate antibiotic use behaviors. The study also identifies specific misconceptions and misuses, such as discontinuing antibiotics when feeling better and using antibiotics without a prescription, which are consistent with findings from other studies in the region. Based on these findings and considering the limitations of this study, including the potential for sampling bias and social desirability bias, we recommend the development and implementation of targeted educational interventions that go beyond simply providing information. These interventions should be designed to inspire and facilitate behavioral change, tailored to specific demographic and cultural contexts within Saudi Arabia, prioritizing engaging, interactive programs that enhance knowledge and promote responsible antibiotic use. Future research should employ more representative sampling strategies, objective measures of behavior, and longitudinal designs to further elucidate the factors driving antibiotic misuse and resistance in the population.
